# Plants as Enrichment? The Effect of Live Plants on the Behavior and Welfare of Indoor‐Housed Titi Monkeys (*Plecturocebus cupreus*)

**DOI:** 10.1002/ajp.70163

**Published:** 2026-05-13

**Authors:** Jon Bunting, Allison R. Lau, Meghan J. Sosnowski, Karen L. Bales

**Affiliations:** ^1^ Department of Psychology University of California Davis California USA; ^2^ California National Primate Research Center University of California Davis California USA; ^3^ Graduate Program in Animal Behavior University of California Davis California USA; ^4^ Department of Neurobiology, Physiology, and Behavior University of California Davis California USA

## Abstract

The biophilia hypothesis states that humans have an innate tendency to enjoy the presence of greenery due to vegetative‐rich areas serving as adaptive environments during our evolutionary history. It is possible that other extant primate species may show a similar response to the presence of plants, but direct experimental interventions on indoor‐housed primates, with plants isolated as a variable, represent a gap in the literature. In this study, 20 coppery titi monkeys (*Plecturocebus cupreus*) housed indoors were exposed to foliage outside of their home cages. Behavior following introduction to plants was recorded and compared to behavior following baseline and control object conditions. The addition of both the plant and control object was associated with a decrease in stereotypic behaviors and an increase in affiliative behavior between bonded mates compared to baseline. The addition of the live plant was associated with increased grooming, and monkeys spent significantly more time looking at the live plant compared to the control object. The reduction in stereotypic behaviors and increase in affiliative behaviors provide support for plants as a potential enrichment strategy. The increase in grooming, combined with greater visual attention to plants, suggests that titi monkeys may have an evolutionary affinity towards plants.

## Graphical abstract

This study tested whether coppery titi monkeys respond positively to plants like humans. The findings show that exposure to live plants reduced stereotypic behaviors, increased affiliative interactions—especially grooming—and attracted more visual attention, suggesting that plants may serve as effective enrichment for titi monkeys and reflect an evolutionary affinity toward greenery.

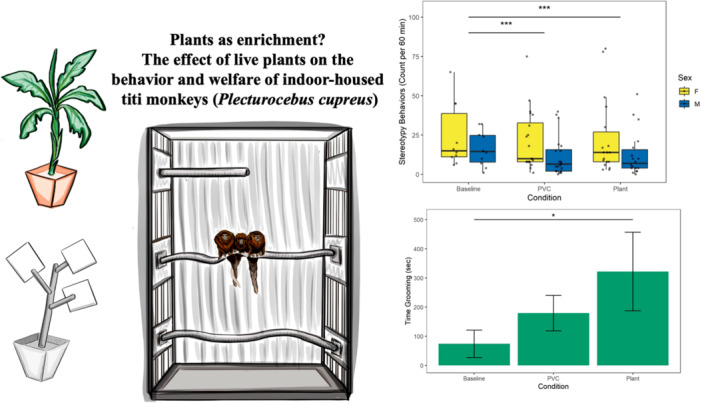

## 1 Introduction

The presence of indoor plants has well‐documented, positive effects on human behavior. A recent review concluded that exposure to plants, be it passive or active, is associated with a variety of beneficial effects, the most prominent being relaxed mood and enhanced cognition (Han et al. 2022). These associations often manifest in short time frames, with effects such as reduced feelings of stress (Lee et al. 2015), reduced illness symptoms (Bjørnstad et al. 2015; Fjeld et al. 1998), and improved cognitive performance (Raanaas et al. 2011) presenting acutely (immediately to 1 day) after exposure, with some evidence suggesting that longer‐term exposure to indoor plants may have effects on health, such as reduced heart rate and blood pressure (Park and Mattson 2008).

These behavioral and physiological changes can be understood through a theory in behavioral biology known as the biophilia hypothesis. This framework asserts that humans possess a biological predisposition to seek connections with living things (Fromm 1973). The biophilia hypothesis, in its current form, describes humans as having a fundamental, evolutionary drive to associate with nature (Wilson 1986; Barbiero and Berto 2021). Though the term biophilia can encompass a desire to engage with both flora and fauna, plants are often the predominant natural feature discussed when considering biophilia in humans, likely because hyper‐urbanized living spaces with low exposure to greenery are a particularly clear example of a disconnect between humans and nature (Grimm et al. 2008). Critics of the biophilia hypothesis have argued that the framework is based on an oversimplified view of human evolution, as well as an overestimation of the degree to which these effects are specific to plants (Joye and De Block 2011). Nonetheless, biophilia has grown into a ubiquitous theory in environmental psychology and has tremendously influenced modern architectural design philosophies (Soderlund and Newman 2015). In the present study, we suggest that a biophilia drive is not unique to modern humans, but is instead a conserved evolutionary preference based upon ecological pressures.

Because this phylogenetic perspective suggests that preferential proximity to plants arose early in the evolutionary history of humans, it is reasonable to suppose that extant primate species may share a similar propensity for plants, given that common ancestors may have faced similar ecological pressures. If nonhuman primates (NHP) show a preference for plant exposure, then plants could provide a simple and cost‐effective enrichment strategy for NHP in captivity. Using plants to improve the welfare conditions of captive NHP is not a novel idea. Some of the existing enrichment literature recommends the addition of plants into enclosures of captive NHP (Coe 1989), but studies establishing a clear preference for plants through experimental interventions are surprisingly limited. Such interventions have been tested more often for distantly related animals, such as for factory farmed fish (Favero Neto and Giaquinto 2020), where the implications of increased physical welfare have obvious fiscal implications, or for amphibians used in biomedical research (Ramos and Ortiz‐Díez 2021). Recent literature suggests that these distant lineages of organisms could have an affinity for greenery similar to humans. For example, *Xenopus laevis* frogs exhibit a preference for enrichment shelters designed to resemble the naturally occurring plant coverage found in their geological habitats compared to the industry standard of polyvinyl chloride (PVC) shelters (Ramos and Ortiz‐Díez 2021). Similarly, the addition of plants to housing tanks is associated with reduced cortisol levels in Zebrafish (*Danio rerio*) (Giacomini et al. 2016), and less stereotypic aggressive behavior in Nile tilapia (*Oreochromis niloticus*) (Favero Neto and Giaquinto 2020).

A few previous existing studies used plants to enrich colonies of captive NHP. West African pottos (*Perodicticus potto*) performed a wider repertoire of behaviors and engaged in mating behaviors for the first time following the addition of grapevines, artificial trees, and bamboo to the enclosure (Frederick and Fernandes 1996). Similarly, cotton‐top tamarins engaged in a wider repertoire of behaviors and exhibited less species‐typical stress behaviors when plants were grown inside their enclosure (Chamove 2005). However, these studies did not isolate the ability to interact with and consume the plants from the effect of the mere visual presence of the plants. As most studies of biophilia in humans have focused on the presence, for true comparison, we would need to assess if changes in behavior are due to expanded availability of space in the cage and new climbing opportunities, as opposed to the visual effect of greenery.

To assess the viability of the biophilia hypothesis in other primates, we studied how the visual presence of plants affected the behavior of captive coppery titi monkeys (*Plecturocebus cupreus*). Coppery titi monkeys are a species of small, arboreal primates endemic to South America (Mason 1966). Titi monkeys are most well known for their enduring pair bonds formed between adults (Díaz‐Muñoz and Bales 2016). A pair bond is defined as a selective psychosocial attachment to another adult individual characterized by preference for one's mate over strangers, distress upon separation, and the ability to buffer one's partner from stress (Bales et al. 2021). Titis are considered a socially monogamous primate (Díaz‐Muñoz and Bales 2016), and consequently, these animals’ unique pair‐bonding behaviors make them a compelling animal model to test the effects of plant introduction into enclosures; titi monkeys’ known pair‐bond behaviors provide a well‐established baseline against which to observe behavioral changes. The colony of titi monkeys at the California National Primate Research Center (CNPRC) lives in indoor enclosures without regular access or visual exposure to greenery, which means they represent a unique opportunity to isolate any behavioral changes in response to the plants to a biological predisposition, as opposed to learned associations from previous experiences with specific plants.

The present study aims to directly observe the impact of novel, passive exposure to plants on the behavior of NHP in order to uncover enrichment implications, and (more broadly) to test the biophilia hypothesis in a new species. To study this, we recorded the social and affective behavior of coppery titi monkey pairs under three conditions: a live plant condition, a control object condition, and a baseline condition. We hypothesized that the introduction of plants would lead to a reduction in stereotypic behaviors, as well as an increase in affiliative behavior between the pairs.

## 2 Methods

### 2.1 Subjects

Ten pairs of adult titi monkeys (10 males, 10 females, *N* = 20 individuals) living at the CNPRC participated in this study. Each pair lived in a 1.2 m × 1.2 m × 2.1 m stainless steel home cage throughout the duration of the study. The number of animals in each cage ranged from 2 to 4 individuals. Each cage at the facility was equipped with five perches, a food bowl, and water access. The temperature of the room was kept at approximately 21°C. Room lighting was maintained at 12‐h light, 12‐h dark, with additional access to natural lighting via skylights. Titi monkeys were fed a standard, species‐appropriate diet of monkey chow, carrots, bananas, apples, and rice cereal twice daily throughout the duration of this study and given *ad libitum* access to water.

This study was approved by the IACUC of the University of California, Davis, and met all legal requirements of the United States as well as guidelines set by the American Society of Primatologists for the ethical treatment of non‐human primates.

### 2.2 Study Design

We recorded the behavior of titi monkey subjects under three conditions. In addition to a baseline recording of the animal's behavior on a typical day with no experimental objects present, each pair was exposed to two experimental conditions: (1) visual exposure to a control object made of PVC piping and (2) visual exposure to a living plant.

**Figure 1:**
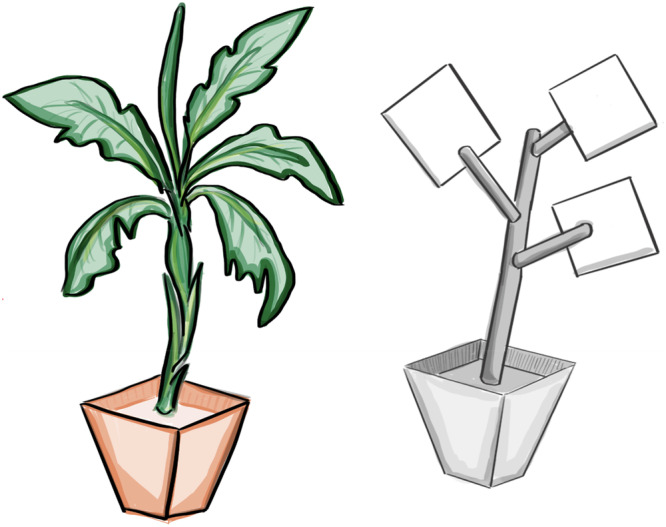
Illustration of the live plant (left) and PVC object (right) presented to coppery titi monkeys (*Plecturocebus cupreus*) in the study.

We chose *Musa acuminata* (Cavendish banana) as the plant for this study; the large leaves and strong green coloration made them an ideal species, as well as the fact that they are frequently found in the animals’ native area of origin of Peru (Staver et al. 2022). Due to its particularly high desiccation tolerance, the plants stayed healthy despite long periods of indoor storage with inconsistent watering. All plants were approximately 1 m × 0.5 m × 1.2 m in size and contained no fruit or flowers. The control objects were constructed out of white PVC, thick gray construction paper, and round nursery planters that matched the plant pots. The size of these objects matched the size of the live plants, approximately 1 m × 0.5 m × 1.2 m (Figure 1).

During all three conditions, two large, suspended curtains were used to block the visual access of non‐study animals housed near the study subjects. These curtains were hung to prevent study subjects from possibly being impacted by the responses of non‐participant animals to the study stimuli. The curtains also ensured subjects were not prematurely exposed to the stimuli during another subject cage's test day. The area of space in front of the cage of interest and between the black curtains took up a footprint of approximately 2 m × 3.5 m. This type of visual barrier (temporary curtains) was familiar to the animals and was held constant across all three conditions. Each condition involved direct behavioral observation of two pairs (*N* = 2) of animals (*N* = 4) in neighboring cages. As titi monkeys are a neophobic species (Mayeaux and Mason 1998), we monitored each session for 15 min to ensure animals did not exhibit signs of excessive stress response.

As no previous studies directly tested the effects of titi monkey habitat enrichment using plants, we modeled our object exposure time periods after biophilia research done on human participants. Specifically, one study determined that 5 h of contact with nature per week coincided with the peak of positive psychological impacts, with no increasing or decreasing impacts as time increased further (White et al. 2019). We opted to expose the titi monkeys to each object for 5 h twice over two consecutive days. Each exposure began at approximately 9:00 h each day and concluded at 14:00 h, resulting in a 19 h gap between exposures. Our criteria for this were to allow the first 5 h of exposure to function as a potential acclimatization period. Baseline recordings were always completed first, and each condition (plant or control object) was always presented over consecutive days, but the order of whether the animals received the plant or control object first was counterbalanced. For example, each group received the same condition on day 2 of exposure as on day 1, followed by 2 consecutive days of the alternate condition 1 week later.

Each session was recorded using a video camera on a tripod that remained in the room for the duration of the session. Behavior was later scored using an ethogram constructed to target behaviors previously established to be indicators of affective arousal and affiliation in this species (Mason and Mendoza 1998; Table 1). Some behaviors were scored on an individual basis, while some were scored as a pair‐level behavioral event. The sex of the animal in the pair was recorded when an individual behavior was scored. *Proximity*, *Contact*, and *Tail Twining* were all mutually exclusive behaviors, meaning one could not be scored at the same time as another. One observer scored all occurrences of behavior over the first hour of each condition using Behavior Tracker software (www.behaviortracker.com).

**Table 1:** Behavioral ethogram of pair‐scored and individual‐scored behaviors.

Behavior	Definition	Individual/Pair	Frequency/Duration
Contact	Two animals are physically touching.	Pair	Duration
Proximity	Animals in arm's reach of one another.	Pair	Duration
Tail twine	Tails of two animals wrapped around each other, with at least one full twine.	Pair	Duration
Grooming	Animals use their hands to pick at each other's fur.	Pair	Duration
Pacing	Repetitive locomotion that follows a set path; at least three repetitions of the same motor path occur.	Individual	Duration
Head twist	Monkey looks up and behind itself in a circular motion.	Individual	Frequency
Self‐scratch	Monkeys scratch any part of their body with their hand or feet.	Individual	Frequency
Tail lash	Tail swings side to side in a lashing motion. Must swing to both sides at least once to score.	Individual	Frequency
Back arch	The animal arches its back, hair stands up on end, and front arms may leave the perch.	Individual	Frequency
Looking at the object	An animal orients one's head toward the object. Eye gaze is trained on the object.	Individual	Duration
Lip smack	The animal moves its face towards the other animal's face and quickly scrunches its lips.	Individual	Frequency

### 2.3 Data Analysis

At the end of data collection, we had a total of 48 observations from 10 monkey groups (one baseline session, two control object condition sessions, and two plant condition sessions per group). Two plant condition observations (one each from two pairs) were missing due to personnel scheduling conflicts, which were accounted for in our modeling analysis. We split the overall dataset into two types of data: pair‐scored and individual‐scored data as described in the ethogram (Table 1), and analyzed the two types of data separately to avoid pseudoreplication of the pair‐level data within the individual‐scored dataset.

For the pair‐scored dataset, which consisted entirely of duration data, we calculated a total contact affiliation rate that was based on the sum of time spent in contact and the sum of time spent tail‐twining (which were scored as separate behaviors in the ethogram). We then constructed two separate linear mixed models (LMMs) in which we predicted the time spent engaged in each distance‐to‐pairmate behavior (total contact affiliation and non‐contact proximity), using condition as a fixed categorical effect, with pair and session number included as crossed random effects. We then compared the two experimental conditions (PVC object and plant) to each other using estimated marginal means with a Satterthwaite degrees‐of‐freedom calculation and a Tukey's *p*‐value correction for multiple comparisons. For all pairwise models, the referent category was the baseline condition.

Upon inspecting the data for grooming duration, we noticed that the dataset was positively skewed and zero‐inflated. Therefore, we accounted for this by constructing a zero‐inflated generalized linear mixed model (GLMM) using a negative binomial distribution predicting grooming duration by condition, with pair and session number as random effects. We otherwise followed the same post‐hoc and model fit procedures as described above for the distance‐to‐pairmate LMMs.

We then used the individual‐scored dataset, which consisted mostly of count data with the exception of object looking time, to assess individual patterns in how monkeys responded to the different conditions, as well as potential sex differences in their response. We began by constructing an LMM in which we assessed how looking time duration differed between the control and plant conditions, and if there was an exposure effect as represented by session number. In this model, we did not include the baseline condition, as there was no object for monkeys to direct their gaze to during the baseline; thus, in this model alone, the referent category was the PVC object condition.

Due to the range of individual differences in how monkeys exhibit potential stress or arousal, we aggregated counts of back arching and tail‐lashing into one measure: “affective arousal.” Similarly, we aggregated counts of head twisting and self‐scratching into another measure: “stereotypic behavior.” We defined stereotypic behavior as repetitive behavior that occurred in response to a stimulus and lacked an obvious goal for function (Mason 2006). We then fit a GLMM using a Poisson family distribution for each measure to assess how the fixed effects of condition and individual sex (as well as the interaction term) influenced the number of instances of affective arousal behaviors or stereotypic behaviors within each session; in these models, we included the crossed random effects of pair and session number. As before, the referent category was the baseline condition, and we compared the two experimental conditions (PVC object and plant) to each other using estimated marginal means.

All statistical analyses were conducted using the R programming language (R Core Team 2022) within RStudio (Posit team 2022). Modeling analyses were conducted using the “lmer” and “glmer” functions of the *lme4* package (Bates et al. 2015) or the “glmmTMB” function of the *glmmTMB* package (Brooks et al. 2017); pairwise comparisons of object conditions used the “emmeans” function of the *emmeans* package (Lenth et al. 2025). Plots were created using the “ggplot” function of the *ggplot2* package (Wickham 2016). We compared each LMM or GLMM to a null model with the same model structure consisting only of the respective intercept and random effects in each model.

## 3 Results

### 3.1 Pair‐Scored Behavior

**Figure 2:**
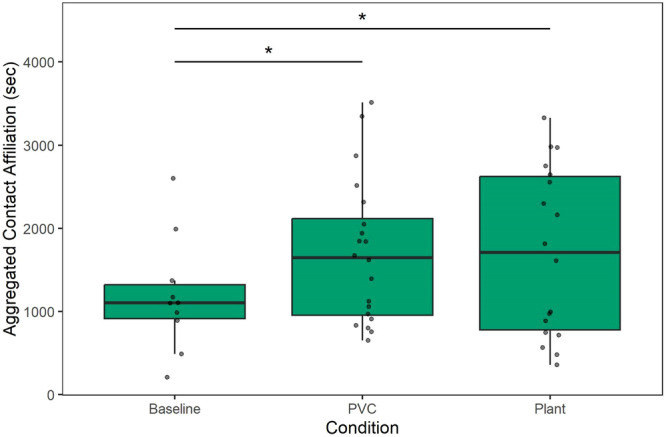
Aggregated contact affiliation by condition out of a total 3600 s of observation. Monkeys spent more time in contact in the PVC control condition (*p* = 0.032) and the plant condition (*p* = 0.046) compared to baseline, but no significant difference was observed between the PVC and plant conditions (*p* = 0.989).

Titi monkeys spent significantly more time in aggregated contact affiliation with their pairmate in the PVC object (*β* = 510.75, SE = 229.77, 95% CI = [47.06–974.44], *t* = 2.22, *p* = 0.032) and the plant (*β* = 481.75, SE = 232.72, 95% CI = [8.06–955.44], *t* = 2.05, *p* = 0.046; Table 2) conditions as compared to baseline. However, there was no difference in contact affiliation between the two object conditions themselves (PVC Object–Live Plant: *β* contrast = 29.0, SE = 201.0, *t* = 0.144, *p* = 0.989), as indicated by post‐hoc contrasts using estimated marginal means (Figure 2). Monkeys did not differ in the amount of time that they spent in non‐contact proximity to their pair mate in either the PVC object (*β* = − 58.65, SE = 51.85, 95% CI = [−163.29 to −45.99], *t* = −1.13, *p* = 0.264) or the plant (*β* = −93.44, SE = 52.85, 95% CI = [−200.10 to −13.22], *t* = −1.77, *p* = 0.084; Table 3) conditions as compared to baseline. In addition, the two experimental conditions did not differ significantly from one another in terms of non‐contact proximity (Control Object–Live Plant: *β* contrast = 34.8, SE = 45.1, *t* = 0.77, *p* = 0.723).

**Table 2:** LMM predicting pair affiliation from condition.

	Contact affiliation			
Predictors	Estimates	Std. error	CI	*p*
(Intercept)	1190.2	272.3	640.69–1739.71	< 0.001
Condition [PVC]	510.75	229.77	47.06–974.44	0.032
Condition [Plant]	481.75	234.72	8.06–955.44	0.046
N _Family_	10			
N _Session_	2			

*Note:* Observations: 48. Marginal *R* ^2^/Conditional *R* ^2^ : 0.106/NA.Full vs. null‐model *χ* ^2^ (2) = 5.21, *p* = 0.074.

**Table 3:** LMM predicting non‐contact proximity from condition.

	Proximity duration			
Predictors	Estimates	Std. error	CI	*p*
(Intercept)	231.8	43.96	143.08–320.52	< 0.001
Condition [PVC]	−58.65	51.85	−163.29 to −45.99	0.264
Condition [Plant]	−93.44	52.85	−200.10 to −13.22	0.084
N _Family_	10			
N _Session_	2			

*Note:* Observations: 48 Marginal R^2^/Conditional R^2^: 0.062/NA Full vs. null‐model *χ^2^* (2) = 2.98, *p* = 0.23.

**Figure 3:**
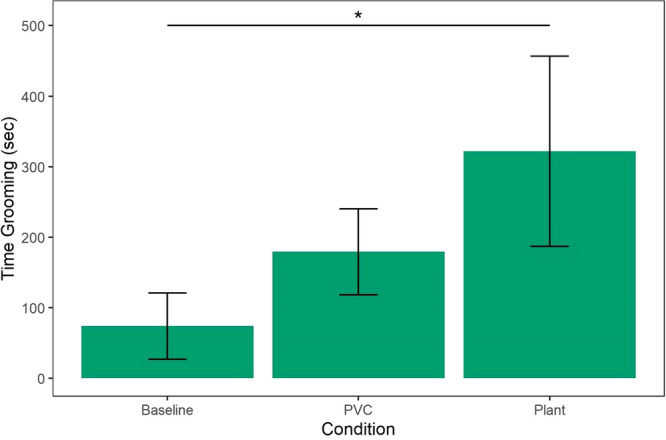
Grooming time (s) by condition out of the total 3600 s of observation. Monkeys spent more time grooming in the plant condition compared to the baseline condition (*p* = 0.015), but not in the PVC condition (*p* = 0.345).

Monkeys did, however, show a difference in whether they groomed and how long they groomed in different conditions. Grooming rates were significantly higher in the plant condition than in the baseline condition (Incidence Rate Ratio = 4.97, SE = 3.26, 95% CI = [1.37–17.97], *z* = 2.44, *p* = 0.015; Table 4), but were not higher in the PVC object condition (Incidence Rate Ratio = 1.76, SE = 1.06, 95% CI = [0.54–5.71], *z* = 0.95, *p* = 0.345); however, the contrast between the two object conditions was not significant (PVC Object–Live Plant: *β* contrast = −1.04, SE = 0.51, *z* = −2.03, *p* = 0.105; Figure 3).

**Table 4:** Zero‐inflated negative binomial GLMM predicting grooming from condition.

	Grooming duration			
Predictors	Incidence rate ratios	Std. error	CI	*p*
(Intercept)	144.49	74.42	52.65–396.49	< 0.001
Condition [PVC]	1.76	1.06	0.54–5.71	0.345
Condition [Plant]	4.97	3.26	1.37–17.97	0.015
N _Family_	10			
N _Session_	2			
Observations	48			

*Note:* Full vs. null‐model *χ* ^2^ (3) = 17.45, *p* < 0.001.

### 3.2 Individual‐Level Behavior

**Figure 4:**
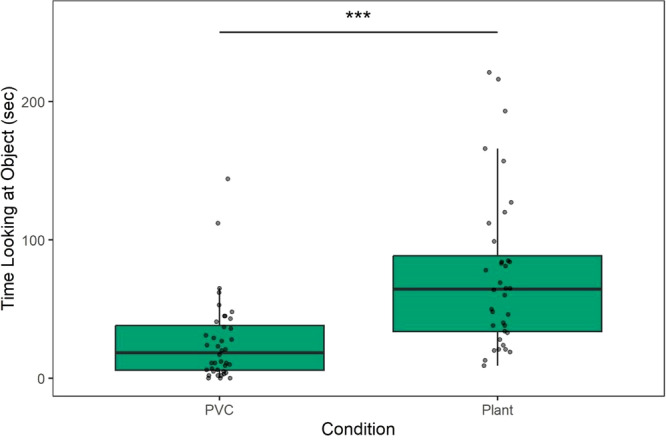
Time looking at the object (s) between conditions out of a total 3600 s of observation. Monkeys looked at the plant significantly longer than the PVC condition (*p* < 0.001).

We first assessed if there was a difference in monkeys’ interest in the plant as compared to the PVC object. Monkeys spent significantly longer looking at the plant as compared to the PVC object (*β* = 46.81, SE = 9.06, 95% CI = [28.75–64.87], *t* = 5.17, *p* < 0.001; Table 5; Figure 4). However, monkeys spent less time looking at either object in the second session as compared to their first exposure to that object (*β* = −32.84, SE = 9.06, 95% CI = [−50.90 to −14.78], *t* = −3.63, *p* = 0.001; Table 5), suggesting that there was an exposure effect on their measured interest.

**Table 5:** LMM predicting looking time (s) from condition and session.

	Looking duration			
Predictors	Estimates	Std. error	CI	*p*
(Intercept)	42.85	8.52	25.85–59.85	< 0.001
Condition [Plant]	46.81	9.06	28.75–64.87	< 0.001
Session [2]	−32.84	9.06	−50.90 to −14.78	0.001
N _Subject_	20			
N _Family_	10			

*Note:* Observations: 76 Marginal R^2^/Conditional R^2^: 0.361/NA Full vs. null‐model *χ^2^* (2) = 32.80, *p* < 0.001.

**Figure 5:**
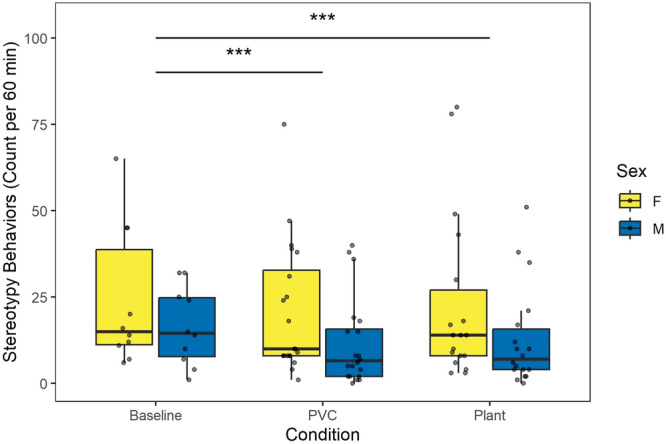
Stereotypic behaviors (head twisting and scratching) as predicted by condition and sex. Across all conditions, females displayed more stereotypic behaviors than males (*p* = 0.018). In response to both the PVC condition (*p* < 0.001) and plant condition (*p* < 0.001), monkeys displayed fewer stereotypic behaviors compared to baseline.

We assessed the prevalence of stereotypic behaviors, a common measure of welfare (Mason 2006), in the presence of the plant and control object compared to baseline. We found that monkeys showed significantly fewer stereotypic behaviors in both the PVC object (Incidence Rate Ratio = 0.65, SE = 0.05, 95% CI = [0.57–0.75], *z* = −5.26, *p* < 0.001) and plant (Incidence Rate Ratio = 0.7, SE = 0.05, 95% CI = [0.61–0.80], *z* = −5.07, *p* < 0.001) conditions as compared to baseline (Table 6). However, there was also a significant main effect of sex, such that males exhibited significantly fewer stereotypic behaviors than females overall (Incidence Rate Ratio = 0.63, SE = 0.12, 95% CI = [0.43–0.92], *z* = −2.37, *p* < 0.001; Figure 5) across all conditions.

**Table 6:** LMM predicting stereotypic behavior from condition.

	Stereotypic behaviors			
Predictors	Incidence rate ratios	Std. error	CI	*p*
(Intercept)	26.13	6.11	16.53–41.32	< 0.001
Condition [PVC]	0.65	0.05	0.57–0.75	< 0.001
Condition [Plant]	0.7	0.05	0.61–0.80	< 0.001
Sex [M]	0.63	0.12	0.43–0.92	0.018
N _Subject_	20			
N _Family_	10			
N _Session_	2			

*Note:* Marginal *R* ^2^/Conditional *R* ^2^: 0.176/0.881 Full vs. null‐model *χ* ^2^ (2) = 32.80, *p* < 0.001

**Figure 6:**
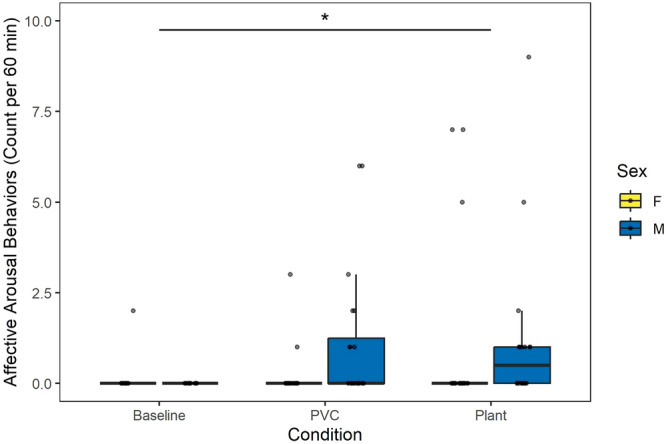
Affective arousal behaviors as predicted by condition and sex during 3600 s of observation. Monkeys displayed more arousal behaviors in response to the plant compared to baseline (*p* = 0.021), but the difference between the PVC condition and baseline was not significant (*p* = 0.099). No sex difference was observed (*p* = 0.18).

We assessed affective arousal behaviors and found that monkeys showed significantly more arousal behaviors in the plant condition (Incidence Rate Ratio = 9.51, SE = 9.27, 95% CI = [1.41–64.29], *z* = 2.31, *p* = 0.021) but not the PVC object condition (Incidence Rate Ratio = 5.23, SE = 5.24, 95% CI = [0.73–37.22], *z* = 1.65, *p* = 0.099) as compared to baseline (Table 7). However, the contrast between the two object conditions was not significant (PVC Object–Live Plant: *β* contrast = −0.60, SE = 0.61, *z* = −0.98, *p* = 0.587). There was no sex difference in affective arousal behaviors (Incidence Rate Ratio = 2.66, SE = 1.95, 95% CI = [0.64–11.16], *z* = 1.34, *p* = 0.18; Figure 6) across all conditions.

**Table 7:** Zero‐inflated negative binomial GLMM predicting affective arousal behaviors from condition.

	Affective arousal			
Predictors	Incidence rate ratios	Std. error	CI	*p*
(Intercept)	0.05	0.05	0.01–0.42	0.006
Condition [PVC]	5.23	5.24	0.73–37.22	0.099
Condition [Plant]	9.51	9.27	1.41–64.29	0.021
Sex [M]	2.66	1.95	0.64–11.16	0.18
N _Subject_	20			
N _Family_	10			
N _Session_	2			

*Note:* Observations: 96 Full vs. null‐model *χ* ^2^ (3) = 8.11, *p* = 0.044.

**Figure 7:**
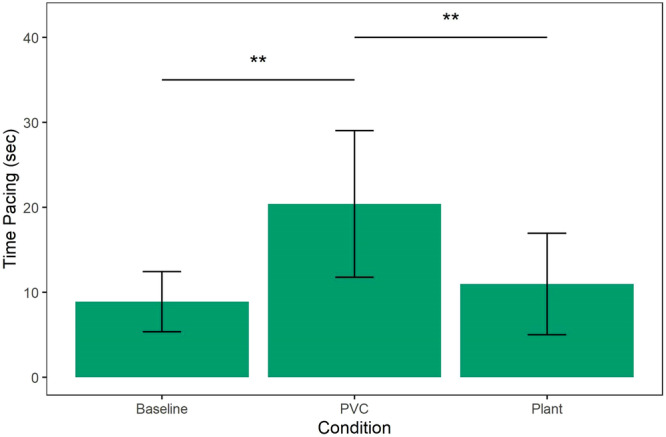
Time spent pacing (s) as predicted by condition out of a total 3600 s of observation. Monkeys spent more time pacing in the PVC condition compared to baseline (*p* = 0.007) and compared to the plant (*p* = 0.001). No significant difference in pacing duration was observed between the plant and baseline conditions (*p* = 0.29).

Finally, we examined pacing duration and found that monkeys paced significantly more in the PVC object condition (Incidence Rate Ratio = 1.59, SE = 0.27, 95% CI = [1.14–2.22], *z* = 2.71, *p* = 0.007) but not the plant condition (Incidence Rate Ratio = 1.2, SE = 0.21, 95% CI = [0.85–1.70], *z* = 1.06, *p* = 0.29) as compared to baseline (Table 8). The contrast between the two object conditions was significant (PVC Object–Live Plant: *β* contrast = 0.28, SE = 0.07, *z* = 3.73, *p* = 0.001; Figure 7) such that more pacing was observed in the presence of the PVC object as compared to the live plant.

**Table 8:** Zero‐inflated Poisson GLMM predicting pacing from condition.

	Pacing duration			
Predictors	Incidence rate ratios	Std. error	CI	*p*
(Intercept)	19.22	8.31	8.23–44.84	< 0.001
Condition [PVC]	1.59	0.27	1.14–2.22	0.007
Condition [Plant]	1.2	0.21	0.85–1.70	0.29
N _Subject_	20			
N _Family_	10			
N _Session_	2			

*Note:* Observations: 96 Marginal *R* ^2^/Conditional *R* ^2^: 0.007/0.357 Full vs. null‐model *χ* ^2^ (2) = 19.15, *p* < 0.001.

## 4 Discussion

After the introduction of both novel objects into the titi monkey's visual field, titi monkeys showed increased affiliation with their pair mate, decreased stereotypic behaviors, and greater affective arousal behaviors compared to baseline. The titi monkeys spent more time looking at the plant compared to the control object. Two of the examined behaviors differed between the PVC object and the plant. First, titi monkeys paced for a greater duration of time when viewing the PVC object compared to baseline (there was no effect of the plant on pacing compared to baseline). Titi monkeys spent more time grooming each other when in the presence of the plant, but not the PVC object, compared to baseline. The results of this study (1) illustrate the potential for visual object interventions as possible enrichment strategies and (2) support the viability of the biophilia hypothesis in titi monkeys. The fact that the majority of our findings are consistent in both the plant and control object conditions confirms the efficacy of object enrichment in this species. The decrease in stereotypic behaviors, compared to baseline, for both conditions, indicates a likely positive impact of the objects on titi monkey welfare.

Our observation that the monkeys spent more time grooming when in the presence of the plant (compared to baseline), but that this effect was not observed in the presence of the PVC object, indicates a potential evolutionary predisposition towards plants. The increased pacing in the presence of the PVC object compared to baseline, but not in the presence of the plant, indicates an increased vigilance or arousal to the PVC object that could be interpreted as slightly less positive than the plant. Finally, the fact that the animals spent significantly more time looking at the live plant relative to the control object allows for a few interpretations. While this higher visual attention to the live plant (vs. the control object) may not be enough evidence to assert that the difference in looking behavior is a result of an evolutionary predisposition towards plants, it illuminates the need for further study. There is also a possibility that the observed increases in contact affiliation (in the presence of both objects) and grooming (in the presence of the plant) compared to baseline are the result of comfort‐seeking behaviors, rather than the objects causing a positive impact on affect.

Because the objects were similar in physical footprint, the factors that contributed to one object being more visually interesting than another were likely a result of shade/color, malleability, or complexity. The plant was naturally more visually complex than the control object, as its surfaces contained a variety of small tears, debris, minor color gradients, and irregularity compared to the smooth surface of the PVC object. There is a possibility that the monkeys perceived the plant as something food‐like, compared to the inedible construction of the PVC object. An interesting future intervention could be a similar experiment with one (or several) intermediate objects with varying degrees of similarity between a live plant and the PVC control object. This could help pinpoint the specific features needed to elicit increased looking behavior. Specifically, constructing a control object with (1) more intricacies than the one used in this study, (2) *no* physical resemblance to a plant, or (3) a green PVC control object, could be useful, as a difference in looking behavior between this proposed object and a live plant could support the idea that plants are specifically and uniquely interesting.

Our findings provide new insight into titi behavior in response to novel stimuli. The increased affiliation in response to both novel objects supports the idea that the items we presented could both be used as enrichment. Increased affiliative behavior is often considered an indication that the pair has a good degree of stability, increasing the likelihood of long‐term success of the animal's relationship, leading to potential positive health effects (Bales et al. 2021). A study with a longer‐term exposure to the objects, on the scales of weeks or months, would be beneficial to further uncover the potential benefits of this form of object enrichment. Alternatively, the increase in affiliative behavior observed in this study could be a comfort‐seeking behavior in response to some aversion to the novel object. While typically we see an increase in high‐arousal behaviors when titi monkeys are distressed, seeking out comfort may be an alternative strategy to mitigate any feelings of aversion to the novel objects.

We observed a reduction in stereotypic behaviors (self‐scratching and head‐twisting combined) compared to baseline, which contradicts the expected behavioral tendencies of this neophobic species who typically respond to novelty with apparently functionless stereotypic behavior. This reduction in stereotypic behaviors suggests either (1) a neutral or positive affective response to the novel stimuli or (2) time spent participating in other, non‐stereotypic vigilance behaviors. While titi monkeys are generally considered a highly neophobic species, under the right circumstances, titis can be highly curious of novel objects (Mayeaux and Mason 1998). This population of titi monkeys may have built a higher‐than‐expected tolerance towards novel stimuli due to the nature of being housed in a research center, where high amounts of novel stimuli (in the form of care staff, equipment, and regular transportation) are an unavoidable aspect of the animals lives. Through this perspective, the novel objects presented in this study could have functioned as a short‐term source of curiosity. Conversely, the reduction in stereotypic behaviors could have been the result of a plant‐specific response to the stimuli (if the titi monkeys assess the plant and PVC control object as equal objects), providing some support for the biophilia hypothesis.

In conclusion, the decrease in stereotypic behaviors, as well as the increase in affiliation and affective arousal within the pair bond, in response to visual exposure to plants and PVC plant‐shaped objects, are promising findings that may support the idea of using plants as an enrichment strategy. The greater visual attention given to the plant compared to the PVC object, as well as the increase in grooming observed in the presence of the plant, support the biophilia hypothesis more generally. Further studies should seek to uncover the specific characteristics of novel objects that are visually salient to titi monkeys, but the present study suggests visual enrichment is indeed a behaviorally salient stimulus to provide to these animals. In the future, carefully introducing a large variety of objects to titi monkeys that vary in their degrees of similarity to real, living plants could be useful in uncovering the specific physical aspects of a plant needed to produce a behavioral change.

## Data Availability

The data that support the findings of this study are available from the corresponding author upon reasonable request.
